# Role and mechanism of alpa1-antitrypsin in polycystic ovary syndrome

**DOI:** 10.15537/smj.2022.43.12.20210398

**Published:** 2022-12

**Authors:** Shouxi Pan, Yilihaer Aizezi, Honglei Wang, Yingli Lu, Baigong Xue

**Affiliations:** *From the Department of Chemistry and Life Science (Pan, Wang), School of Chemistry and Life Science, Changchun University of Technology; from the Department of Cell Biology (Pan, Aizezi, Xue), Norman Bethune College of Medicine, Jilin University; and from the Department of Reproductive Medical Center (Lu), The Second Hospital of Jilin University, Reproductive Medical Center, Changchun, China.*

**Keywords:** Polycystic ovary syndrome, Pro-inflammatory cytokines, Alpha 1-antitrypsin, neutrophil elastase, IL-8, IL-1β, molecular docking

## Abstract

**Objectives:**

To determine the relationships among alpha1-antitrypsin (A1AT), pro-inflammatory cytokines, neutrophil elastase (NE), interleukin (IL)- 1β, and IL-8 in cases with polycystic ovary syndrome (PCOS).

**Methods:**

Female rats in the control group were fed and watered normally. Female rats in the PCOS group were given high-fat diets and letrozole (1% carboxymethyl cellulose’ [CMC] solution) CMC by gavage at a dose of l mg/kg body weight daily for 23 days. The mRNA levels of A1AT and NE in rat ovaries were detected by performing real-time polymerase chain reaction (PCR) in the laboratory of Norman Bethune College of Medicine, Jilin University, China in 2017. All serum samples were collected from the Second Hospital of Jilin University from October 2021 to November 2021 for obesity concurrent with PCOS. Molecular docking of A1AT with NE, IL-8, and IL-1β was investigated using the Insight II software ZDOCK tool. This study was carried out at the Reproductive Center, The Second Hospital of Jilin University, Changchun, China from June 2021 to July 2022.

**Results:**

The expression of the A1AT mRNA decreased in the ovary tissues of PCOS rats relative to that of healthy controls, while the expression of NE mRNA increased compared to that of normal controls. The serum A1AT expression in PCOS cases decreased considerably relative to their expression in normal controls. However, the expression of NE, IL-1β, and IL-8 increased significantly relative to their expression in the control (*p*<0.05 for all). The Insight II ZDOCK molecular docking simulations showed that A1AT has direct interaction sites for NE, IL-1β, and IL-8.

**Conclusion:**

Alpha1-antitrypsin is closely associated with NE, IL-1β, and IL-8. Therefore, we speculate that A1AT might ameliorate PCOS symptoms by inhibiting pro-inflammatory factors: NE, IL-1β, and IL-8.

**P**olycystic ovary syndrome (PCOS) is a female endocrine disorder associated with metabolic and reproductive dysfunction.^1,2^ In many patients with PCOS, obesity or being overweight is the major factor leading to anovulatory infertility.^3^ Polycystic ovary syndrome starts to develop at the onset of increased gonadal and adrenal activity around puberty, and both gonadarche and PCOS reflect functional changes in the hypothalamic-pituitary-ovarian axis.^4^ Its exact etiology and pathogenic mechanism are not known, and it continues to present a complex challenge in the field of gynecological endocrinology. Many studies on the condition have shown that pro-inflammatory cytokines have critical effects on the pathogenic mechanism of PCOS, while autoimmune inflammation might also underly it.^5,6^ Inflammation theory, and its interaction with PCOS, is a highly important aspect of research, shaping the current perspective that PCOS is a chronic inflammatory disorder.

Alpha1-antitrypsin (A1AT) is an important serine protease inhibitor in humans. It is mainly synthesized in hepatic cells, and its level in circulation increases considerably in inflammatory disorders.^7,8^ Alpha1-antitrypsin is not only a glycoprotein found in the acute phase; but also a critical neutrophil protease inhibitor, protecting tissues against protein hydrolysis injury under various inflammatory conditions.^9,10^ As PCOS is often accompanied by obesity, hyperinsulinemia, and insulin resistance, inflammation might be a mechanism related to its development.^11^ Obesity is a common comorbidity complicating PCOS, and studies have found that overweight PCOS female patients exhibit a high risk of anovulation and abnormal menstruation compared to women of normal weight: human serum A1AT levels decrease as BMI increases.^12^ The anti-inflammatory properties of A1AT have been widely reported in clinical application, but few have been reported in female reproductive disorders.

In this study, we determined the relationship between A1AT and pro-inflammatory cytokines, solely among PCOS patients also developing comorbid obesity, by analyzing variations in the neutrophil elastase (NE), A1AT, interleukin (IL)-1β, and IL-8 levels. We also investigated the A1AT-related mechanism in PCOS to identify effective targets for the treatment of PCOS.

We randomly assigned 20 rats (8 normal rats and 12 PCOS rats) into 2 groups. The rats in the PCOS group were given letrozole (1% carboxymethyl cellulose [CMC] solution) CMC by a high-fat diet, combined with gavage at l mg/kg body weight for 23 days.^13^ All experiments were performed following the National Institutes of Health guidelines for the care and use of animals.

All serum samples were collected from 40 cases (average age: 32.8±3.1 years) with normal blood glucose levels, who visited The Second Hospital of Jilin University, Changchun, China from October 2021 to November 2021 for obesity concurrent with PCOS. The diagnostic criteria of PCOS have not been standardized due to the heterogeneous nature of PCOS. Polycystic ovary syndrome was diagnosed from the following aspects: i) The presence of symptoms from puberty, ii) menstrual irregularities, iii) chronic anovulation, iv) hirsutism, v) high blood LH (luteinizing hormone) or the LH/follicle-stimulating hormone ratio, combined with excess testosterone levels, vi) ultrasound examination of polycystic ovarian symptoms to exclude similar diseases

Obesity was diagnosed in PCOS cases whose body mass index (BMI) was ≥30 kg/m^2^. Each subject excluded pulmonary, hepatic, renal, and cardiovascular diseases, family or genetic history of tumors, and cardiopulmonary and renal dysfunction.

Additionally, 50 healthy individuals participated in the study as normal controls. This study was approved by the Ethics Committee of The Second Hospital of Jilin University and was carried out following the guidelines of the Declaration of Helsinki. Each participant provided informed consent before participation.

## Methods

In this study, 20 female rats were randomly divided into 2 groups, 10 each in the control group and the PCOS group. Female rats in the control group were fed and watered normally. Female rats in the PCOS group were given high-fat diets and letrozole (1% CMC solution) CMC by gavage at a dose of 1 mg/kg body weight per day. After 23 days of continuous administration, vaginal exfoliated cells lost their periodicity in the PCOS state. Rat ovarian tissue total ribonucleic acid (RNA) was isolated, which was prepared into first-strand complementary deoxyribonucleic acid (cDNA) through reverse transcription, followed by quantification through reverse transcription polymerase chain reaction (RT-PCR). The results were used to determine the difference in the NE and A1AT messenger RNA (mRNA) levels between PCOS and normal control groups and investigate how A1AT and NE are associated with the development of PCOS. The A1AT and NE mRNA levels in rat ovaries were detected by performing RT-PCR in the laboratory at Norman Bethune College of Medicine Jilin University in 2017.

### Statistical analysis

Fasting venous blood (5 mL) was extracted in the early morning, preserved for 30 min (minutes) at 37°C, and then centrifuged at 3,000 rpm for 15 min. The expression of serum A1AT, NE, IL-1β, and IL-8 was determined by performing ELISA. The ELISA kit was purchased from Millipore Corporation (Billerica, MA), and the assay was performed following the manufacturer’s instructions. The data were recorded using the HDM_9602G ELISA analyzer (Plantier Technology Co., Ltd., Beijing, China). The Statistical Package for the Social Sciences, version 16.0 (SPSS Inc., Chicago, IL) software was used for data processing, with statistical significance determined at *p*<0.05. The relationships between A1AT and variable values, as well as the relationship between A1AT and NE, were made for Spearman analysis.

Molecular docking of A1AT with NE, IL-8, and IL-1β was simulated using the insight II 2005 ZDOCK software docking program. The operating parameters were as follows: RMSD cutoff=6, angular step size=6, interface cutoff=9, top poses=2,000, and maximal cluster number=60. The complexes with the highest RDOCK scores were optimized with the RDOCK module. The interactions between residues on the interfaces of the obtained complexes were calculated using the LigPlot+ v.1.4 software (UCL Business PLC [UCLB]; London, UK). This study was conducted at the Reproductive Center, The Second Hospital of Jilin University, Changchun (China), from June 2021 to July 2022.

## Results

**Figure 1:**
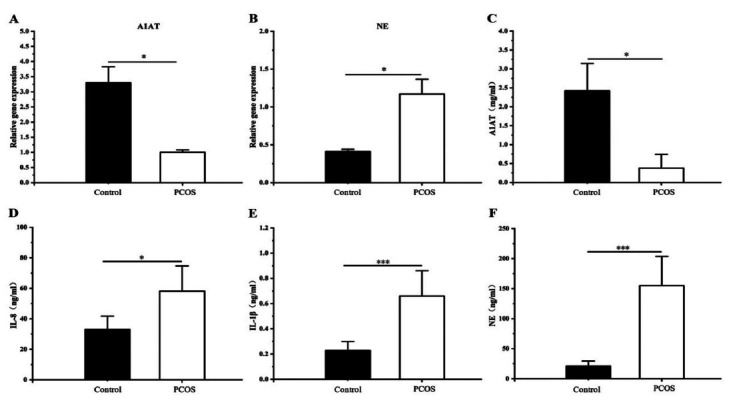
- Expression of tissue and serum A1AT, NE, IL-1β, and IL-8 levels between polycystic ovarian syndrome (PCOS) cases and normal controls. **A-B**: The relative alpha1-antitrypsin (A1AT) and neutrophil elastase (NE) mRNA levels in the ovaries of PCOS and normal control rats. **C-F**: Serum A1AT, interleukin (IL)-1β, IL-8, and NE expression in PCOS cases and controls. *p<0.05, **p<0.01, ***p<0.001

The results of the RT-PCR analysis showed that the A1AT and NE levels in the ovaries of PCOS rats differed significantly from those in normal controls (NCs). The relative A1AT levels in the ovaries of the PCOS rats were significantly lower than those in NCs, as determined by RT-PCR analysis (*p*<0.05, Figure 1A). The relative NE expression in the ovaries of PCOS rats was significantly higher than that in normal controls (*p*<0.05, Figure 1B).

Alpha1-antitrypsin levels among PCOS cases differed significantly compared to those in normal controls, and the results of ELISA showed that A1AT levels in the peripheral blood of PCOS cases were significantly lower than those in NCs (*p*<0.05, Figure 1C). The expression of serum IL-8 was significantly higher in PCOS patients (*p*<0.05, Figure 1D), and IL-1β was also significantly higher in PCOS patients (*p*<0.001, Figure 1E). Serum levels of NE, one of the most important substrates of A1AT, were higher in PCOS patients, and the NE/A1AT ratio was also higher, which was significantly different from that of normal controls (*p*<0.001, Figure 1F).

**Figure 2:**
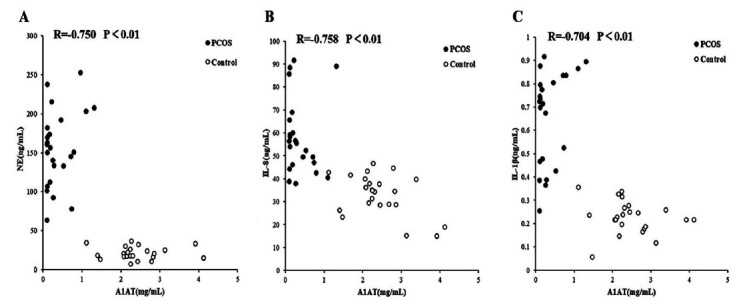
- Comparison of serum A1AT, NE, interleukin (IL)-1beta, and IL-8 levels between polycystic ovarian syndrome cases and normal controls.

Serum NE, IL-1β, and IL-8 expressions were negatively related to the A1AT expression among PCOS cases. The A1AT expression was negatively related to NE (R= -0.750, *p*<0.01). The A1AT was negatively related to IL-8 (R= -0.758, *p*<0.01). The A1AT was negatively related to IL-1β (R= -0.704, *p*<0.01). Neutrophil elastase was positively related to IL-8 (R=0.787, *p*<0.01). The expression of NE was positively related to IL-1β (R=0.826, *p*<0.01). The expression of IL-8 was positively related to IL-1β (R=0.744, *p*<0.01). The results are presented in Table 1 and Figure 2.

**Table 1:** - Analysis of the correlation between various factors and alpha1-antitrypsin (A1AT).

Factors	A1AT	IL-8	IL-1β	NE
A1AT	1	-0.758**	-0.704**	-0.750**
IL-8	-0.758**	1	0.744**	0.787**
IL-1β	-0.704**	0.744**	1	0.826**
NE	-0.750**	0.787**	0.826**	1

IL-1β: interleukin-1beta, NE: neutrophil elastase

**Figure 3:**
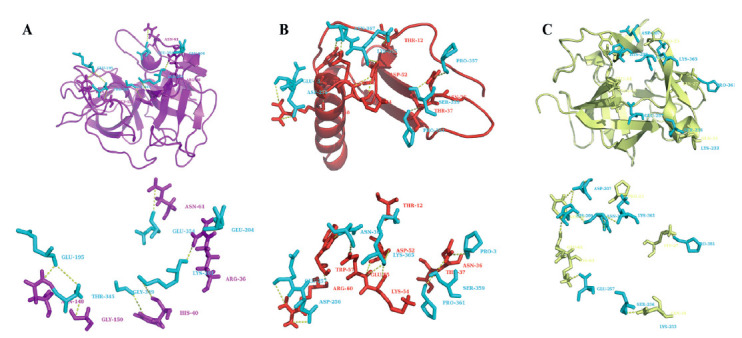
- The residues involved in polar interactions are labeled and shown in the stick renders. The interactions between residues are shown in yellow dashed lines. **A**) The complex of NE (purple) and alpha1-antitrypsin (A1AT) (blue). **B**) The complex of interleukin (IL)-8 (red) and alpha1-antitrypsin (blue). **C**) The complex of IL-1β (yellow) and A1AT (blue).

The Insight II ZDOCK molecular docking simulations showed that A1AT has direct interaction sites for NE, IL-1β, and IL-8. The simulated molecular structural docking of A1AT with NE, IL-1β, and IL-8 was performed to investigate the interaction of A1AT with pro-inflammatory factors NE, IL-1β, and IL-8. The crystal structure data were obtained from the Protein Data Bank (PDB) database (A1AT PDB ID: 1KCT; IL8 PDB ID: 3IL8; IL-1β PDB ID: 2I1B; NE PDB ID: 3Q76). The molecular docking calculations were performed using the ZDOCK module of the Insight II software. Each couple of complexes was calculated by the ZDOCK module to obtain 2,000 conformations. The complexes with the highest RDOCK scores were selected as the final results by optimizing the RDOCK module of the Insight II software. As shown in Figure 3, the docking results indicated that the active central residue of A1AT can be combined with the active sites for pro-inflammatory factors NE, IL-1β, and IL-8 to form many polar interactions. Furthermore, the residues PRO361 and LYS365 in the loop of the A1AT active center were involved in the formation of polar intermolecular interactions between the 2 complex molecules, indicating that these 2 residues were essential for A1AT to inhibit IL-1β and IL-8.

**Figure 4:**
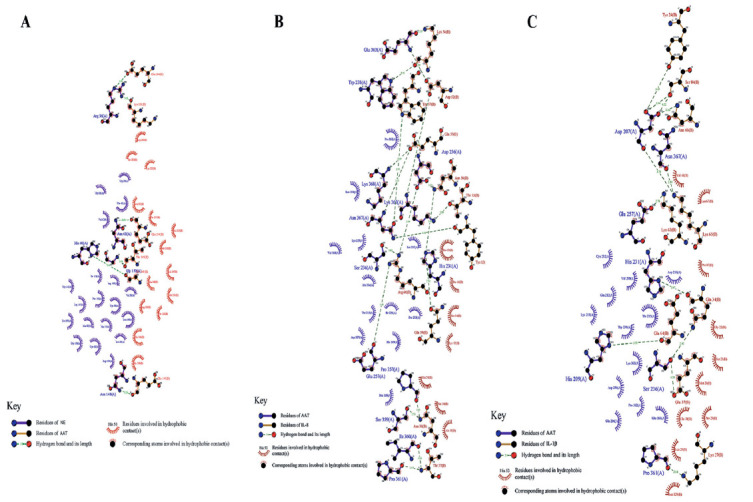
- Calculated interactions between the residues on the interfaces of the neutrophil elastase and alpha1-antitrypsin (A1AT) complex, the interleukin (IL)-8 and A1AT complex, and the IL-1β and A1AT complex.

The complexes obtained by molecular docking were analyzed using the LigPlot software to further investigate the key residues of A1AT that interact with NE, IL-1β, and IL-8. As shown in Figure 4B, besides forming polar interactions, the complexes of A1AT and IL-8 have many residues involved in the formation of hydrophobic intermolecular interactions (A1AT: PRO362, LEU254, LYS259, VAL364, SER237, ALA284, THR215, ILE229, PRO255, ASP207, HIS209, and MET358; IL-8: PRO16 PRO53, SER14, LYS15, GLU29, SER30, and ALA35). These polar and hydrophobic interactions have critical effects on stable complex formation. Both the A1AT/IL-1β complex and the A1AT/NE complex also form many polar and hydrophobic intermolecular interactions (Figure 4 A-C). A1AT binds to NE, IL-1β, and IL-8, while inhibiting their activities through their direct interactions with active site residues.

## Discussion

Obese patients often exhibit metabolic abnormalities, oxidative stress, and chronic low-grade inflammation, all of which increase pro-inflammatory factors, such as monocyte neutrophil chemotactic factor protein (MCP-1) and tumor necrosis factor (TNF-α), while decreasing anti-inflammatory factors such as adiponectin. The levels of the pro-inflammatory factors MCP-1 and TNF-α were significantly lower in normal individuals than that in PCOS cases.^14,15^ Obese patients with PCOS often have chronic adipose inflammation and insulin resistance. Chronic low-grade adipose tissue inflammation might also be mechanistically associated with metabolic disorders and complications of organ tissues among obese and overweight individuals. The interaction of various pro-/anti-inflammatory signals greatly increases the difficulty in treating PCOS.^16^ Leptin levels are high in obese individuals, and adipose inflammation is associated with leptin resistance. Leptin is negatively correlated with A1AT, and an increase in leptin levels inhibits A1AT expression, exacerbating — along with the inflammatory response — the lower A1AT levels in obese patients.^17,18^ However, the complications associated with molecular events for the initiation of immune cell infiltration in adipose tissue, which in turn affect the production of inflammatory cytokines, are unknown.^19^ Adipose tissue is a metabolic endocrine organ with high activity. It strongly influences PCOS progression by secreting pro-inflammatory factors.^20^ Therefore, in this study, we only investigated obese PCOS patients, who inherently have more adipose tissue.

A macrophage is a type of immunocyte. It is most abundant in adipose tissues, and it strongly affects the inflammation of these tissues. Due to their sensitivity to the surrounding environment, macrophages possess various phenotypes: namely anti-inflammation and pro-inflammation. In healthy adipose tissues, macrophages are polarized to the anti-inflammatory M2 type to maintain adipose tissue homeostasis. However, in obese adipose tissue, macrophages are polarized to the pro-inflammatory M1 type; these changes induce the production of pro-inflammatory factors, which impairs insulin signaling and promotes insulin resistance.^21^

Impairment of macrophage mitochondria leads to an obesity-mediated inflammatory response in macrophages, alongside systemic insulin resistance. Mitochondrial dysfunction induces IL-1β release, thus decreasing insulin sensitivity in the insulin-targeting cells through paracrine signaling and their infiltration in circulation. Insulin resistance and the associated hyperinsulinemia can induce the endocrine and reproductive features of PCOS.^22^

Obesity is accompanied by a decrease in A1AT expression. A1AT can be generated by the liver to inhibit NE, while NE represents the neutrophil protease produced during inflammation.^23^ Such an imbalance in A1AT-NE affects inflammatory responses. An increase in the NE/A1AT ratio increases the production of inflammatory factors. Neutrophil elastase causes the accumulation of pro-inflammatory cytokines and decreases the ratio of NE to A1AT, thus inhibiting the accumulation of pro-inflammatory factors.^24,25^ PCOS cases showed higher levels of pro-inflammatory factors than normal controls, indicating the critical effect of the increase in the levels of pro-inflammatory factors on the pathogenic mechanism of PCOS^26^. These findings reflect those of similar studies. The A1AT mRNA levels in the ovaries of PCOS rats were lower than those in the ovaries of normal controls, while the expression of NE mRNA was higher than that of normal controls. Polycystic ovary syndrome cases showing comorbid obesity had significantly lower A1AT levels and significantly higher NE levels. An increase in the NE/A1AT ratio might contribute to the accumulation of pro-inflammatory factors, such as IL-1β and IL-8. This, in turn, might be associated with the pathogenic mechanism of PCOS.

Alpha1-antitrypsin can also reduce the levels of pro-inflammatory factors such as NE, IL-1β, and IL-8. In a rat model of peritoneal infiltration, Lewis et al^27^ found that A1AT not only inhibited neutrophil migration by inhibiting IL-8 secretion by leukocytes, but it also inhibited the migration and aggregation of macrophages, thus impeding immune infiltration of the graft. For alleviating acute inflammation in pancreatic island transplantation, Koulmanda et al^28^ studied gene expression in lymph nodes using real-time quantitative fluorescence PCR and found that A1AT inhibited the synthesis of acute-phase reactants and pro-inflammatory factors (such as IL-1, IL-6, TNF, IFN-g, and TNF-α), increased the levels of anti-inflammatory factors, and altered the immune balance in pancreatic lymph nodes.

The results of molecular docking showed that A1AT has direct interaction sites for NE, IL-1β, and IL-8: such as A1AT binds to and directly interacts with NE, IL-1β, and IL-8 through the active sites of the residues and inhibits the pro-inflammatory factors NE, IL-1β, and IL-8. By suppressing the expression of NE, the ratio of NE to A1AT can be further decreased along with the levels of the pro-inflammatory factors IL-8 and IL-1; this, in turn, can improve the therapeutic effects on PCOS.

### Study limitations

Polycystic ovary syndrome treatment has not yet been standardized since the pathogenic mechanism of PCOS remains unknown. The treatment of PCOS is usually selected based on clinical presentation and expected outcomes. In this study, we determined the link between low-grade chronic inflammation among obese PCOS patients and the pathogenic mechanism of PCOS. Alpha1-antitrypsin levels are low in obese PCOS patients, and A1AT deficiency might lead to the accumulation of pro-inflammatory factors, including NE, IL-1β, and IL-8, which are involved in the PCOS-related pathogenic mechanism.

In conclusion, A1AT might improve PCOS treatment by inhibiting pro-inflammatory factors NE, IL-1β, and IL-8 while improving the therapeutic outcome for PCOS.

Further studies need to determine whether A1AT can alleviate the symptoms of PCOS by improving excess androgen production, mitigating menstrual disorders, mitigating metabolic diseases, resisting endometrium, and enhancing fertility. Targeting A1AT to alleviate low-grade chronic inflammation among PCOS patients to improve their condition might be effective and represents a significant advancement in the treatment of PCOS. Thus, this study provided a new strategy for treating PCOS.
